# Influence of myrtle extract supplementation via drinking water on performance, blood hematology, biochemistry, and intestinal morphology in Wistar albino rats

**DOI:** 10.3389/fvets.2026.1770774

**Published:** 2026-02-25

**Authors:** Ümit Özçınar, İsmail Bayram, Ali Çalık, Mehmet Fatih Bozkurt, Muhammet Emre Orman, Eyüp Eren Gültepe, Mustafa Midilli, Syed Rizwan Ali Shah, Mudassar Zafar, Barış Denk, İsmail Hakkı Özsandık, İbrahim Sadi Çetingül

**Affiliations:** 1 Department of Animal Nutrition and Nutritional Diseases, Faculty of Veterinary Medicine, Afyon Kocatepe University, Afyonkarahisar, Türkiye; 2 Department of Animal Nutrition and Nutritional Diseases, Faculty of Veterinary Medicine, Ankara University, Ankara, Türkiye; 3 Department of Pathology, Faculty of Veterinary Medicine, Afyon Kocatepe University, Afyonkarahisar, Türkiye; 4 Department of Poultry Science, Faculty of Agriculture and Nature Science, Abant İzzet Baysal University, Bolu, Türkiye; 5 Department of Biochemistry, Faculty of Veterinary Medicine, Afyon Kocatepe University, Afyonkarahisar, Türkiye

**Keywords:** antioxidant, cytokine, glucose, metabolism, *Myrtus communis*

## Abstract

This study aimed to evaluate the effects of *Myrtus communis* extract added to drinking water on performance parameters, blood physiology, selected biochemical parameters, and small intestinal histomorphology in rats. A total of 80 healthy 30-day-old Wistar albino rats (40 female and 40 male) were randomly assigned to control or treatment groups, each further divided into eight subgroups. The experimental groups received *Myrtus communis* extract in drinking water at concentrations of 0% (control), 2.5, 5, 7.5, and 10% for a period of 35 days. Body weight and feed intake were recorded weekly, while water consumption was measured daily. At the end of the experiment, all animals were anesthetized and euthanized; blood samples were collected by cardiac puncture, liver tissues were sampled for cytokine and heat shock protein analyses, and small intestinal tissues were collected for histopathological evaluation. Supplementation with *Myrtus communis* extract did not affect the body weight, water consumption and feed consumption. While the serum glucose level was lower in the 2.5% group; addition of the extract at the 10% concentration decreased the serum urea and blood urea nitrogen levels. The blood physiological parameters were not influenced by the treatment except for increased basophil counts in the treatment groups. Expression levels of proinflammatory cytokines, HSP70 and HSP90 were similar among the groups. The treatment significantly increased the villus length, crypt depth and Proliferating Cell Nuclear Antigen (PCNA) scores in the small intestine. In conclusion, supplementation of *Myrtus communis* extract via drinking water improved intestinal morphology and epithelial proliferative activity and modulated serum glucose levels, while exerting limited effects on systemic inflammatory markers and performance parameters. These findings suggest that *Myrtus communis* may serve as a functional phytogenic additive supporting intestinal health without compromising growth performance.

## 1 Introduction

Increasing awareness of potential health and safety concerns together with a growing interest in natural, plant-derived resources has stimulated research on alternative approaches aiming to support animal health and physiological function (1). Increasing regulatory restrictions on the use of certain feed additives and antibiotics, driven by health and safety concerns, have stimulated research into natural, plant-derived alternatives (2).

Previous studies have reported that herbal products (extracts, essential oils, and powders) have positive impacts on the immune system and antioxidant capacity of the host (3, 4). The reasons for these positive effects include findings that they increase the retention and digestibility of nutrients, increase the secretion of digestive enzymes (5) promote mucus production (6), show antiviral and antioxidant properties, activate the immune response, and have anthelmintic properties (7).

The myrtle (*Myrtus communis* L.), which is an evergreen plant in the family Myrtaceae, is native to the Indian Subcontinent, Southern Europe, North Africa, and Western Asia, whereas it can also be grown in other regions (8). Recent studies have reported that *Myrtus communis* exhibits several biological activities, including antioxidant, antimicrobial, and anti-inflammatory effects, which are largely attributed to its bioactive ingredients such as tannins, alkaloids, flavonoids and oils (9, 10). Myricetin-3-O-galactoside and myricetin-3-O-rhamnoside, which are compounds isolated from the leaves of *Myrtus communis*, have been reported to inhibit the activity of xanthine oxidase, reduce lipid peroxidation, and scavenge free radicals such as 1,1-diphenyl-2-picrylhydrazyl (11).

Aggul et al. (12) demonstrated that high-dose ethanolic extracts of *Myrtus communis* fruits effectively controlled hyperglycemia and enhanced antioxidant defense in a diabetic rat model. Similarly, Hassan et al. (13) reported that myrtle plant extract exerted protective effects against hepatic disorders associated with monosodium glutamate and acrylamide reducing their cytotoxic effects in rats. In a study by Safari et al. (14), it was shown that myrtle could significantly improve mucosal immunity, antioxidant activity, and growth and appetite genes in zebrafish at the molecular level. Additionally, myrtle oils and extracts have been investigated across various animal species including broilers, laying hens, rabbits, rats, and quail to enhance performance and physiology (15–18).

Despite extensive reports on the biological activities of *Myrtus communis*, no studies, to our knowledge, have examined the effects of administering an extract prepared using through drinking water in rats. In particular, studies addressing its effects on intestinal morphology together with heat shock protein expression and cytokine responses in mammalian models are scarce. Therefore, this study aimed to evaluate the effects of *Myrtus communis* extract supplementation via drinking water on performance, as well as intestinal morphology, heat shock protein expression, cytokine profiles, and selected blood physiological and biochemical parameters in rats. It was hypothesised that *Myrtus communis* extract could enhance physiological homeostasis by modulating metabolism, intestinal morphology, and inflammatory responses, while also providing a scientific basis for future studies in other animal species.

## 2 Materials and methods

This study was approved by the Afyon Kocatepe University Animal Experiments Local Ethics Committee on 14/04/2021 with the decision number 49533702/48.

The study was carried out in the Experimental Animals unit of the Faculty of Veterinary Medicine at Afyon Kocatepe University. The animal material of the study included a total of 80 weaned Wistar albino rats, with an average age of 30 days. The animals were divided into 5 groups (4 treatments, 1 control), consisting of 16 animals in each group. Each group was divided into 8 subgroups (4 subgroups of females, 4 subgroups of males), each with 2 animals (2 males or 2 females) by random sampling. While no intervention was applied to the feed or drinking water of the control group, the drinking water of the experimental groups was supplemented with 2.5, 5, 7.5, and 10% myrtle extract, respectively. At the beginning of the study, a ten-day acclimatization period was allowed. The trial continued for 35 days. A lighting schedule providing 12 h of light and 12 h of darkness was adopted. All groups were fed ad libitum rat pellet feed during the study. The Weende analysis method was used to determine the nutrient content of the pellet feed given to the rats. The nutrient composition of the rat feed included 90.83% dry matter, 24.75% crude protein, 4.50% crude cellulose, 1.90% crude fat and 8.16% ash on a dry matter basis.

The myrtle extract added to the drinking water of the groups was kept at an average room temperature. The animals were weighed weekly to monitor their growth. At the end of the experiment, blood, liver, and small intestine tissue samples were collected from all animals in each group under xylazine and ketamine anesthesia. The collected tissue samples were stored in a formaldehyde solution in the refrigerator for later pathological examinations. The blood samples were collected into tubes with and without anticoagulants. Serum and plasma were extracted from the blood samples by centrifugation at 5000 rpm for 10 min. Complete blood count and serum biochemistry analyses were performed immediately.

### 2.1 Extract production, validation, and composition of extract

The active ingredients of the *Myrtus communis L.* extract are shown in Table 1. The extraction processes for the Myrtus plant were carried out by Bioderm®, ArsArthro Biotechnologies Inc. in Ankara, Türkiye, following the methods outlined by Handa et al. (19). Extraction was performed using dried leaves and bark in distilled water (1% *Myrtus communis* extract and 99% distilled water) as the only solvent.

**Table 1:** Active ingredients of the *Myrtus communis* L plant extract.

Amino acid	(mg/L)	Mineral	(mg/L)
Alanine	691.27	Lead (Pb)	0.007
Arginine	634.54	Cadmium (Cd)	0.002
Aspartic Acid	668.01	Mercury (Hg)	0.03
Cystine	218.82	Arsenic (As)	0.003
Glutamic Acid	1767.92	Sodium (Na)	324,000
Glycine	417.49	Magnesium (Mg)	27,000
Histidine	1133.84	Aluminum (Al)	1.35
Isoleucine	414.90	Iron (Fe)	7.1
Leucine	173.50	Tin (Sn)	1
Lysine	0.00		
Methionine	281.53	Chemical Composition	(mg/L)
Proline	16254.60	Myricetin	15.34
Serine	182.91	Catechin	4.80
Threonine	0.00	Quercetin	0.19
Tryptophan	907.13	Gallic acid	0.13
Valine	151.64	Salicylic acid	0.06
Tyrosine	1003.04	Rosmarinic acid	0.01
Phenylalanine	895.06		
Norvaline	72.46		

The active ingredient composition of the extract was analyzed using liquid chromatography and mass spectrometry (LC–MS/QTOF) (Orbitrap). An Agilent Poroshell 120 ECC183.0 × 100 mm, 2.7 μm column was used with a mobile phase containing 10 mM ammonium formate and 0.1% formic acid. All components exhibited both negative and positive polarization. Amino acid contents were determined using the high-performance liquid chromatography (HPLC) technique based on Brückner and Westhauser’s method (20), while mineral contents were analyzed using the Shimadzu ICPMS-2030 device.

### 2.2 Total RNA isolation, cDNA synthesis and quantitative real-time PCR

Liver tissues were sampled, quickly frozen in liquid nitrogen, and stored at −86 °C until RNA extraction. Approximately 20–30 mg of tissue was weighed, and the total RNA was isolated using a NucleoSpin RNA kit. The concentration and purity of the RNA were assessed with a NanoDrop 2000 spectrophotometer. For cDNA synthesis, 2 μg of RNA was used with the One Script® Plus kit, and the resulting cDNA was stored at −20 °C. The mRNA quantities of Heat Shock Proteins (HSP 70 and HSP 90) and cytokines (IL-1β, IL-10, and TNF-*α*) were measured using the CFX Connect Real-Time Polymerase Chain Reaction (PCR) System (Bio-Rad) and the Blas Taq 2X qPCR Master Mix. cDNA samples were diluted 1:5 with molecular water before the RT-PCR, which included 10 μL of the Master Mix (Table 2), 1 μL each of forward and reverse primers, 4 μL of molecular water, and 4 μL of diluted cDNA. The assay was initiated with an activation step at 95 °C for 3 min, followed by 40 cycles consisting of denaturation at 95 °C for 15 s, and combined annealing and extension at 60 °C for 1 min. The specificity of the generated products was confirmed by melting curve analysis. GAPDH was used as the reference gene for normalization. Relative mRNA expression was calculated using the 2^-ΔΔCt method, where the calibrator sample was defined to have the mean ΔCt value of the control group (21).

**Table 2:** Sequences of primer pairs used for amplification of target and reference genes.

Gene^1^	Primer sequence	Size	Acc (Reference)
HSP70	ACGAGGGTCTCAAGGGCAAG	107	NM_031971.2
	CTCTTTCTCAGCCAGCGTGTTAG		
HSP90	ACCCAAGACCAACCAATGGA	154	NM_175761.2
	TATCCAGAGCGTCTGAGGAGT		
IL-1β	TCTCACAGCAGCATCTCGAC	177	NM_031512.2
	CATCATCCCACGAGTCACAG		
IL-10	CTGGCTCAGCACTGCTATGT	86	NM_012854.2
	GCAGTTATTGTCACCCCGGA		
TNFα	ACACACGAGACGCTGAAGTA	118	NM_012675.3
	TCCACTCAGGCATCGACATT		
	GGCACAGTCAAGGCTGAGAATG		
GAPDH	GGCACAGTCAAGGCTGAGAATG	143	NM_017008.4
	ATGGTGGTGAAGACGCCAGTA		

^1^IL: Interleukin, TNFα: Tumor necrosis factor alpha, GAPDH: Glyceraldehyde 3-phosphate dehydrogenase. For each gene, the primer sequence for forward (F) and reverse (R) (5’-3’) primers, the amplicon size (bp), and the NCBI accession number (Acc) used for the primer design are listed.

### 2.3 Histomorphological examinations

For histological evaluation, the jejunum was identified, and a 1-cm tissue segment was collected approximately 7 cm proximal to the cecum–ileum junction. The collected samples were gently rinsed with normal saline to remove luminal contents and immediately fixed in 10% neutral buffered formalin for 48 h. Following fixation, tissues were routinely processed, dehydrated, cleared, and embedded in paraffin. Serial sections of 4 μm thickness were obtained using a rotary microtome and mounted on normal and adhesive-coated glass slides.

The sections were stained with hematoxylin and eosin (H&E) and examined under a light microscope (Zeiss Axio Lab. A1 microscope equipped with an Axio Cam ICC 5 camera). Histomorphometric measurements were performed using ImageJ software (National Institutes of Health, United States). Villus height was measured from the tip of the villus to the villus–crypt junction, while crypt depth was measured from the base of the crypt to its transition zone with the villus (22).

### 2.4 Proliferating cell nuclear antigen staining

Paraffin-embedded sections mounted on adhesive-coated slides were processed for immunohistochemical examination of proliferating cell nuclear antigen (PCNA), a well-established marker of cell proliferation, using the avidin–biotin complex (ABC) method. Following deparaffinization and rehydration through graded alcohols, endogenous peroxidase activity was blocked by incubation in 3% hydrogen peroxide (H₂O₂) for 10 min at room temperature. Antigen retrieval was performed by microwave heating in 0.01 M citrate buffer (pH 6.0) for 20 min.

Following antigen retrieval, the sections were allowed to cool and were incubated with blocking serum at 37 °C for 15 min to reduce nonspecific binding. The sections were then incubated with a rabbit polyclonal anti-PCNA primary antibody (ab18197, Abcam, UK; dilution 1:400) at 37 °C for 2 h. Detection was carried out using an ABC detection kit (TA-125-UDX, Thermo Scientific), with 3-amino-9-ethylcarbazole (AEC; TA-060-HA) as the chromogen. The sections were counterstained with Mayer’s hematoxylin, mounted, and examined under a light microscope.

For quantitative analysis, 100 epithelial cells were counted in five randomly selected, well-oriented villi per sample. Cells showing distinct nuclear staining were considered PCNA-positive, and the results were expressed as the percentage of PCNA-positive cells (23).

### 2.5 Statistical analyses

A randomized block design was utilized in the study, with cages serving as the experimental units. Statistical analyses were conducted using PROC MIXED in SAS (SAS Institute Inc., 2008). Dietary treatments were defined as fixed effects, while the cage or animal was considered a random effect. Normality was assessed, and outliers were excluded if the studentized residuals were smaller than −4 or greater than 4. Degrees of freedom were estimated using either the Kenward-Roger or the Satterthwaite equations (24). The Tukey–Kramer adjustment was applied for multiple comparisons. For repeated measures, an AR(1) covariance structure was implemented. Specific contrasts were made to compare the control group to the treatments and evaluate linear and quadratic trends across different levels of supplementation. Contrast coefficients were calculated using PROC IML (25). Data are presented as least square means ± pooled standard errors of the mean (SEM). Statistical significance was set at *p* ≤ 0.05, while trends were noted for *p*-values between 0.05 and 0.15.

## 3 Results

No significant effects of the treatments were found on performance indicators such as daily weight gain, the feed conversion ratio (FCR), and feed consumption. Feed consumption increased over time in all groups (*p* <0.0001). Additionally, the comparisons of water intake between the control and experimental groups revealed linear (*p* = 0.1302) and quadratic (*p* = 0.1375) effects (Table 3). The myrtle extract led to an increases in leading higher consumption in all treatment groups compared to the control group (*p* = 0.0404).

**Table 3:** Effects of different ratios of myrtle supplemented to drinking water on performance of rats for 5 weeks.

Item	Treatment						*p*-values			Contrasts			
	Control	2.5% Myrtus extract	5% Myrtus extract	7.5% Myrtus extract	10% Myrtus extract	SEM	Treatment	Time	Time x treatment	Control vs. Treatment	Linear	Quadratic	Cubic
Feed consumption, g	19.47	19.19	19.45	20.06	20.02	0.4755	0.5963	<0.0001	0.4992	0.9828	0.2878	0.4783	0.3288
Water consumption, ml	25.80	28.00	28.72	28.59	28.15	1.0807	0.3248	<0.0001	0.0067	0.0404	0.1302	0.1375	0.7304
BWG^2^, g	58.67	58.05	58.00	60.50	57.25	3.3927	0.9698	<0.0001	0.3689	0.9531	0.9705	0.8329	0.5597
FCR^3^, g feed/g BWG	4.78	4.95	5.25	5.06	5.22	0.2267	0.5592	<0.0001	0.0057	0.1797	0.1695	0.5338	0.7617

^1^Data are represented as least squares. The values are means of 8 replicate cages per diet with 2 rats (*n* = 16). Control: plain water; 2.5%; 1.25 mL/day/individual myrtle extract was adjusted to a total volume of 50 mL; 5%; 2.5 mL/day/individual myrtle extract was adjusted to a total volume of 50 mL, 7.5%; 3.75 mL/day/individual myrtle extract was adjusted to a total volume of 50 mL; 10%; 5 mL/day/individual myrtle extract was adjusted to a total volume of 50 mL. ^2^Body weight gain. ^3^Feed conversion ratio. ^4^Standard error of the mean. Significance levels were *p* <0.05 for main effects and 0.05 <*p* <0.15 for trends. Coefficients of contrast for treatment levels were calculated using PROC IML in SAS.

The extract had no significant impact on blood biochemistry parameters including the levels of ALT, IgG, and phosphorus. However, significant effects of the extract were noted in glucose (*p* = 0.005), bilirubin (*p* = 0.008), blood urea nitrogen (BUN) (*p* = 0.022), urea (*p* = 0.017), and aspartate aminotransferase (AST) (*p* <0.001) levels. The incorporation of myrtle extract in drinking water by 2.5% resulted in the lowest blood glucose levels among the groups. In contrast, the 2.5% myrtle extract led to the highest levels of bilirubin, urea, and BUN. Myrtle supplementation had a quadratic effect on urea, and BUN levels, (*p* = 0.072, and *p* = 0.058, respectively) (Table 4).

**Table 4:** Effects of different ratios of myrtle supplemented to drinking water on blood biochemistry of rats for 5 weeks^1^.

Item	Treatment						*p-values*	Contrasts			
	Control	2.5% Myrtus extract	5% Myrtus extract	7.5% Myrtus extract	10% Myrtus extract	SEM^4^		Control *vs.* Treatment	Linear	Quadratic	Cubic
Glucose	149.68^ab^	106.61^b^	122.62^ab^	134.12^ab^	156.43^a^	9.865	0.005	0.079	0.194	0.001	0.127
Cholesterol	60.08	58.37	56.69	57.40	52.12	2.803	0.354	0.215	0.062	0.653	0.501
T. Protein	6.20	6.56	6.42	6.47	6.46	0.103	0.183	0.021	0.195	0.172	0.1956
Urea	51.41^a^	53.82^a^	50.54^a^	48.70^a^	41.74^b^	2.522	0.017	0.340	0.003	0.072	0.945
BUN^2^	24.02^ab^	25.15^a^	23.95^ab^	22.76^ab^	19.44^b^	1.207	0.022	0.379	0.004	0.058	0.959
IgG^3^	179.00	211.88	222.33	207.00	184.43	20.378	0.516	0.234	0.926	0.079	0.815
Creatinine	0.23	0.26	0.24	0.21	0.24	0.012	0.104	0.756	0.429	0.970	0.009
Biluribin	0.04^ab^	0.06^a^	0.05^ab^	0.03^b^	0.03^b^	0.006	0.008	0.825	0.016	0.091	0.017
AST^4^	90.58^b^	96.17^a^	81.25^c^	92.67^b^	84.58^c^	8.100	<0.0001	0.6930	<0.0001	<0.0001	<0.0001
ALT^5^	31.83	29.50	33.50	31.67	32.42	12.870	0.995	0.998	0.987	0.999	0.970
Phosphorus	18.59	19.76	19.18	18.80	19.44	0.558	0.603	0.263	0.676	0.679	0.130

^1^Data are represented as least squares. The values are means of 8 replicate cages per diet with 2 rats (*n* = 16). Control: plain water; 2.5%; 1.25 ml/day/individual myrtle extract was adjusted to a total volume of 50 mL; 5%; 2.5 ml/day/individual myrtle extract was adjusted to a total volume of 50 mL, 7.5%; 3.75 mL/day/individual myrtle extract was adjusted to a total volume of 50 mL; 10%; 5 mL/day/individual myrtle extract was adjusted to a total volume of 50 mL. ^2^Blood Urea Nitrogen. ^3^Immunoglobulin G. ^4^Aspartate amino transferase. ^5^Alanine amino transferase. ^6^Standard error of mean. ^a,b,c^Values with different superscripts in the same row are significantly different (*p* ≤ 0.05) and (*p* ≤ 0.01). Significance levels for trends are 0.05 <*p* <0.15. Coefficients of contrast for treatment levels were calculated using PROC IML in SAS.

Additionally, MCH and MCHC values were higher in the treatment groups compared to the control group (*p* = 0.009 and *p* = 0.017, respectively). Similarly, platelet counts were higher in the 5 and 10% groups compared to the control group (Table 5). No significant effect of the extract was found on the other blood physiological parameters.

**Table 5:** Effects of different ratios of myrtle supplemented to drinking water on blood hemogram of rats for 5 weeks^1^.

Hemogram	Treatment						*p*-values	Contrasts			
	Control	2.5% Myrtus extract	5% Myrtus extract	7.5% Myrtus extract	10% Myrtus extract	SEM		Control *vs.* Treatment	Linear	Quadratic	Cubic
RBC^2^, 10^6^/dL	7.65	7.75	7.68	7.65	7.77	0.148	0.968	0.721	0.779	0.886	0.514
MCV^3^, fL	59.33	60.27	59.51	59.86	59.80	0.486	0.700	0.331	0.726	0.626	0.404
MCH^4^, pg	17.65^b^	18.08^a^	17.83^ab^	18.14^a^	18.20^a^	0.138	0.030	0.009	0.009	0.735	0.315
MCHC^5^, g/dL	29.76^b^	29.99^ab^	29.95^ab^	30.32^a^	30.45^a^	0.150	0.011	0.017	0.001	0.679	0.958
WBC^6^, 10^9^/L	5.59	5.87	6.52	6.01	5.90	0.629	0.883	0.491	0.699	0.415	0.988
Neutrophils %	0.86	0.69	0.91	0.82	0.80	0.134	0.803	0.813	0.703	0.775	0.577
Lymphocytes, %	4.31	4.58	5.08	4.83	4.60	0.520	0.873	0.431	0.618	0.376	0.905
Monocytes, %	0.35	0.51	0.43	0.29	0.35	0.071	0.221	0.581	0.318	0.330	0.053
Basophils, %	0.008^b^	0.018^a^	0.019^a^	0.017^a^	0.017^a^	0.002	0.014	0.001	0.031	0.018	0.102
Eosinophils, %	0.04	0.05	0.06	0.04	0.04	0.007	0.263	0.311	0.833	0.054	0.674
Hemoglobin, g/dL	13.50	14.00	13.69	13.89	14.14	0.248	0.387	0.124	0.142	1.000	0.269
Hematocrit, %	45.35	46.73	45.70	45.80	46.45	0.792	0.723	0.357	0.611	0.915	0.240
Platelets, 10^9^/L	821.00^b^	842.17^b^	887.92^a^	834.92^b^	888.83^a^	96.750	<0.0001	<0.0001	<0.0001	<0.0001	<0.0001
PDW^7^, %	8.15	8.35	8.09	7.85	8.06	0.140	0.165	0.700	0.136	0.924	0.042
MPV^8^	7.90	7.83	7.68	7.56	7.58	0.103	0.093	0.047	0.008	0.604	0.507
P-LCR^9^, %	9.08^a^	9.56^a^	8.08^b^	7.25^b^	7.45^b^	0.618	0.045	0.172	0.007	0.970	0.133
PCT^10^, %	0.69	0.65	0.67	0.62	0.67	0.029	0.596	0.331	0.475	0.414	0.656
RDW-CV^11^, %	14.45	13.79	14.35	13.37	13.96	0.431	0.405	0.235	0.315	0.552	0.798
RDW-SD^12^, %	25.42	25.22	25.53	24.50	24.50	0.420	0.452	0.467	0.282	0.903	0.415
NRBC^13^, %	0.01	0.01	0.01	0.01	0.00	0.004	0.790	0.844	0.445	0.380	0.627

^1^Data are represented as least squares. The values are means of 8 replicate cages per diet with 2 rats (*n* = 16). Control: plain water; 2.5%; 1.25 mL/day/individual myrtle extract was adjusted to a total volume of 50 mL; 5%; 2.5 mL/day/individual myrtle extract was adjusted to a total volume of 50 mL, 7.5%; 3.75 mL/day/individual myrtle extract was adjusted to a total volume of 50 mL; 10%; 5 mL/day/individual myrtle extract was adjusted to a total volume of 50 mL. ^2^Red blood cell count. ^3^Mean corpuscular volume. ^4^Mean corpuscular hemoglobin. ^5^Mean corpuscular hemoglobin concentration. ^6^White blood cell count. ^7^Platelet distribution width. ^8^Mean platelet volume (MPV). ^9^Platelet-large cell ratio (P-LCR). ^10^Procalcitonin (PCT). ^11^Red Cell Distribution Width (RDW-CV). ^12^Red Cell Distribution Width standard deviation (RDW-SD). ^13^Nucleated red blood cells (NRBC) ^14^Standard error of mean. ^a,b,c^Values with different superscripts in the same row are significantly different (*p* ≤ 0.05) and (*p* ≤ 0.01). Significance levels for trends are 0.05 <*p* <0.15. Coefficients of contrast for treatment levels were calculated using PROC IML in SAS.

**Figure 1:**
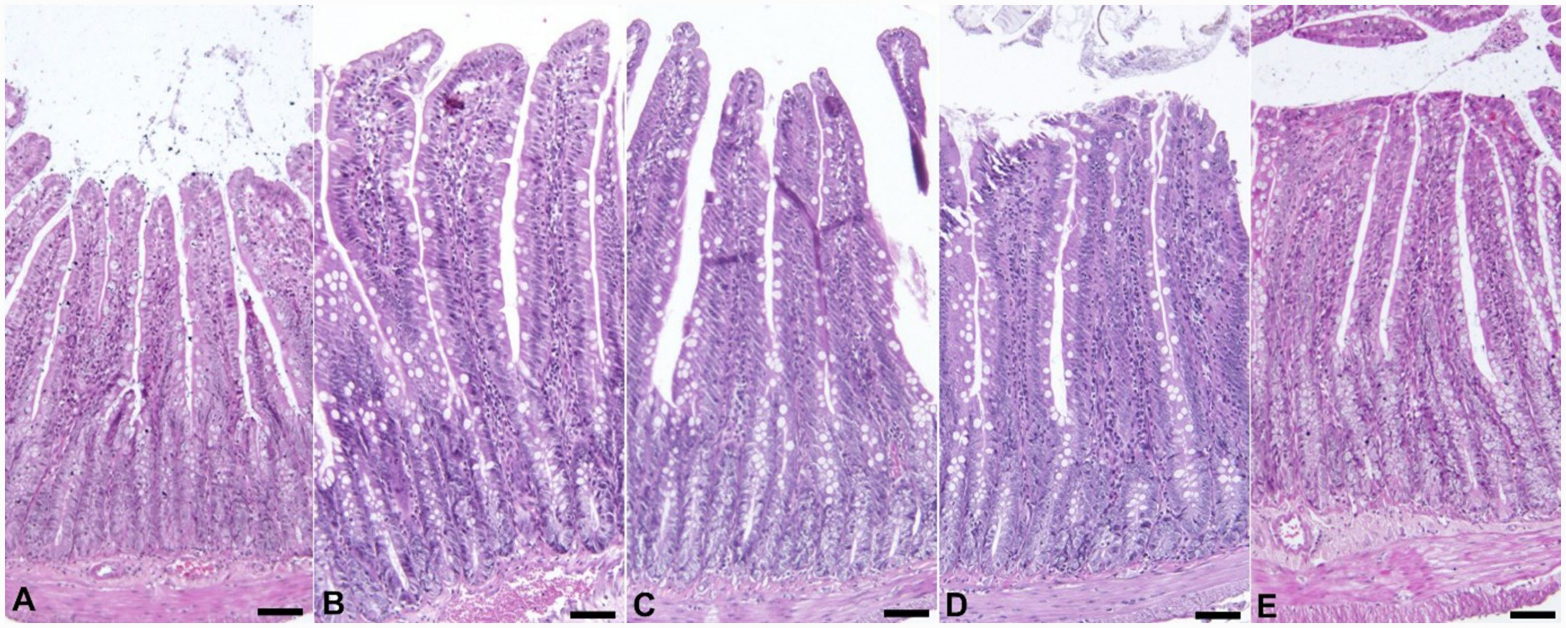
Microscopic view of the jejunum. Shorter villi are seen in the control group **(A)** compared to the other groups **(B–E)**. HE. Bar = 150 μm.

**Figure 2:**
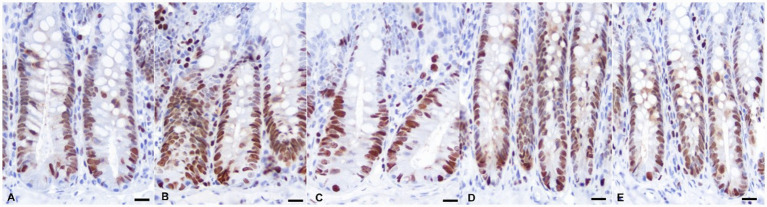
Microscopic image of the PCNA immunohistochemical staining of the jejunum. More intense PCNA positivity (nuclear red color indicates positivity) is seen in group 2 **(B)** and group 4 **(D)** compared to groups 1, 3, 5 **(A, C, E)**. ABC-peroxidase technique, AEC chromogen, Gill’s (III) hematoxylin. Bar = 37 μm.

There was no significant difference among the groups in terms of expression levels of immune system-related cytokines (IL-1β, IL-10, and TNF-*α*), HSP70 and HSP90 in the liver (Table 6). The histomorphological findings are summarized in Table 7, and representative microscopic images of all experimental groups are shown in Figures 1, 2. The addition of myrtle extract increased both villus length and crypt depth in the small intestines of all experimental groups compared to the control group (*p* <0.0001). The maximum villus length was observed in the 2.5% group, while the greatest crypt depth was found in the 10% group. The PCNA values were not affected by the treatment, however, the 2.5 and 7.5% groups had higher expression levels of PCNA.

**Table 6:** Effects of different ratios of myrtle supplemented to drinking water on cytokines and heat shock protein expression of rats for 5 weeks^1^.

Item	Treatment						*p*-values	Contrasts			
	Control	2.5% Myrtus extract	5% Myrtus extract	7.5% Myrtus extract	10% Myrtus extract	SEM^4^		Control *vs.* Treatment	Linear	Quadratic	Cubic
HSP70	1.09	1.06	1.29	1.40	1.39	0.138	0.190	0.190	0.040	0.640	0.300
HSP90	1.10	1.03	0.88	0.93	0.92	0.098	0.490	0.150	0.140	0.400	0.950
IL-1	1.08	1.26	1.24	1.23	1.31	0.183	0.930	0.390	0.460	0.780	0.620
IL-10	1.11	1.20	1.58	1.43	1.40	0.192	0.420	0.170	0.180	0.280	0.780
TNF-ALPHA	1.04	1.12	1.03	1.19	1.27	0.109	0.480	0.360	0.130	0.530	0.790

^1^Data are represented as least squares. The values are means of 8 replicate cages per diet with 2 rats (*n* = 16). Control: plain water; 2.5%; 1.25 mL/day/individual myrtle extract was adjusted to a total volume of 50 mL; 5%; 2.5 mL/day/individual myrtle extract was adjusted to a total volume of 50 mL, 7.5%; 3.75 mL/day/individual myrtle extract was adjusted to a total volume of 50 mL; 10%; 5 mL/day/individual myrtle extract was adjusted to a total volume of 50 mL. ^2^Standard error of mean ^a,b,c^Values with different superscripts in the same row are significantly different (*p* ≤ 0.05) and (*p* ≤ 0.01). Significance levels for trends are 0.05 <*p* <0.15. Coefficients of contrast for treatment levels were calculated using PROC IML in SAS.

**Table 7:** Effects of different ratios of myrtle supplemented to drinking water on villus length, crypt depth, and PCNA values in small intestines of rats for 5 weeks^1^.

Item	Treatment						*p*-values	Contrasts			
	Control	2.5% Myrtus extract	5% Myrtus extract	7.5% Myrtus extract	10% Myrtus extract	SEM^2^		Control *vs.* Treatment	Linear	Quadratic	Cubic
Villus length	346.95^c^	439.64^a^	397.76^b^	395.64^b^	399.56^b^	7.1918	<0.0001	<0.0001	0.0180	<0.0001	<0.0001
Crypt depth	206.34^c^	221.57^b^	231.26^ab^	228.24^ab^	236.85^a^	3.7425	<0.0001	<0.0001	<0.0001	0.0644	0.1479
PCNA score	50.81^b^	59.80^a^	48.42^b^	60.78^a^	48.15^b^	1.1238	<0.0001	0.0075	0.2367	<0.0001	0.2059

^1^Data are represented as least squares. The values are means of 8 replicate cages per diet with 2 rats (*n* = 16). Control: plain water; 2.5%; 1.25 mL/day/individual myrtle extract was adjusted to a total volume of 50 mL; 5%; 2.5 ml/day/individual myrtle extract was adjusted to a total volume of 50 mL, 7.5%; 3.75 ml/day/individual myrtle extract was adjusted to a total volume of 50 mL; 10%; 5 ml/day/individual myrtle extract was adjusted to a total volume of 50 mL. ^2^Standard error of the mean. Significance levels are *p* <0.05 for main effects and 0.05 <*p* <0.15 for trends. Coefficients of contrast for treatment levels were calculated using PROC IML in SAS.

## 4 Discussion

The evaluation of performance indicators in rats receiving different concentrations of myrtle extract in their drinking water showed no significant treatment-related effects excluding increased water intake. However, because the animals were in the growth phase, marked time-dependent increases in these parameters were observed across all groups (26). The water intake increased in treatment groups compared to control, but feed intake was similar in presented study. One of the drivers of the water intake in rats is the flavor of water. It has been shown that the fluid intake is stimulated by the palatability of drinking water (27). For this reason the observed increase in water intake in rats receiving *Myrtus communis* extract is likely related to alterations in the taste or palatability of the drinking water caused by the extract’s bioactive compounds without affecting the feed intake. Similar to present study, Bulbul et al. (28) reported that the incorporation of 5% myrtle plant (Myrtus) oil into the diet of laying quails did not significantly affect their feed intake. However, research conducted by Gultepe et al. (18) revealed that incorporation of myrtle extract into the drinking water enhanced feed and water intake in older laying hens. This discrepancy could result from species-spesific differences in feed intake regulation and physiological status. Water intake is closely associated with feed intake, and phytogenic additives that enhance water palatability often stimulate overall feed consumption in poultry (29, 30). Conversely, feed intake is more tightly regulated by metabolic homeostasis and is largely determined by the animal’s energy requirements (31). The myrtus extract did not affect the body weight gain and feed conversion ratio in rats. In contrast to the present findings, previous study in calves have reported increases in both body weight gain and feed intake following Myrtus supplementation (32). These differences may be related to species-specific physiology, metabolic demands, and the route of administration.

Myricetin, an active constituent of *Myrtus communis*, is a hexahydroxyflavonol, and a considerable proportion of myricetin is absorbed in the intestines of monogastric animals (33). Myricetin has been reported to enhance glucose transport in rat adipocytes without affecting insulin receptor activity or the translocation of glucose transporter type 4 (GLUT-4). Consistent with this mechanism, Ong and Khoo (34) demonstrated that myricetin significantly reduced hyperglycemia in diabetic rats. The results of the present study indicated a reduction in serum glucose levels in rats receiving *Myrtus communis* extract at a concentration of 2.5%; however, the comparison between the control and treatment groups revealed no statistically significant difference. Although *Myrtus communis* has been reported to exert hypoglycaemic effects through multiple mechanisms, these effects appear to be more pronounced in diabetic or hyperglycaemic animal models (35). Since no experimental intervention was applied to induce diabetes or hyperglycaemia in the present study, the hypoglycaemic potential of the extract may not have been fully demonstrated. Notably, the decrease observed in the 2.5% group may suggest that low-dose supplementation has the potential to influence glucose regulation even in normoglycaemic animals. In the present study, no significant differences in blood urea levels were observed between the control and treatment groups overall; however, rats in the 10% group exhibited a statistically significant reduction in blood urea concentration. The significant reduction in blood urea observed in the high-dose *Myrtus communis* group in the present study is consistent with previous reports showing that dietary inclusion of *Myrtus communis* berries can lower blood urea levels without adversely affecting hepatic or renal parameters, possibly due to the polyphenolic constituents of the plant, which may bind dietary proteins (36). Collectively, these findings suggest a dose-dependent modulatory effect of *Myrtus communis* on protein and nitrogen metabolism.

The blood basophil counts increased in treatment groups while other leukocyte counts remained unaffected which may reflect a mild and selective immunomodulation (37). Also, there were no changes in the expression of HSP70, HSP90 and proinflammatory cytokines. Previous studies showed reductions in pro-inflammatory cytokines in disease-induced models (38, 39), whereas a study conducted without experimental disease induction have shown increased TNF-*α* expression in tissues such as the brain and intestine (14). In the present study, no alterations were observed in IL-1, IL-6, TNF-α, HSP70 and HSP90 levels in liver tissue, suggesting that *Myrtus communis* did not cause a systemic response in healthy rats, and that its immunomodulatory effects could be limited to specific tissues. This situation may reflect a mild and targeted immunological modulation rather than generalized immune activation.

In the present study, *Myrtus communis* supplementation significantly increased villus length, crypt depth, and PCNA expression in the intestinal tissue, indicating enhanced mucosal development and epithelial cell proliferation. Similarly, it has been reported that dietary myrtle powder or oil intake increases the villus length (8, 40). The beneficial effects of myrtle and various herbal products have been reported by promoting gut microbial balance, especially increasing the number of colonies of Lactobacilli and Bifidobacteria (41). The probiotic bacteria have a beneficial effect on the intestinal mucosa protecting the intestinal barrier and activating the intestinal epithel proliferation (42). In our study we did not perform any bacterial measurement, however, the improvements in the intestinal morphology could result from potential increase in probiotic bacteria as suggested in previous studies. In addition, beneficial effects of phenolic compounds on the tissue regeneration have been documented (43). The increased expression of PCNA which is an indicator of cell proliferation (44) in treatment groups could suggest that myrtus extract has an stimulatory effect on the intestinal cell proliferation. However, effect of *Myrtus communis* on the cell proliferation should be further investigated.

The present study was conducted in healthy rats under non-challenged conditions, which may have limited the manifestation of systemic metabolic and immunological responses to *Myrtus communis* supplementation. Although, its lowering effect on the blood glucose could make it an functional food additve for diabetic pet animals it should be further investigated in diabetic animals. Consequently, although local intestinal improvements were evident, broader physiological effects may not have been fully expressed. In addition, the absence of molecular analyses such as intestine microbial population classification restricts mechanistic interpretation of the observed intestinal remodeling. In particular, the potential influence of *Myrtus communis* on protein metabolism, intestinal function and tissue remodeling warrants further investigation. Incorporating molecular markers of intestinal proliferation, barrier function, and immune regulation, would further clarify its potential to better define its potential use as a functional phytogenic additive in animal nutrition.

## 5 Conclusion

Consequently, myrtle extract incorporated into drinking water did not affect performance parameters. Although the higher dose of myrtus extract reduced the blood urea nitrogen levels, it reduced the blood glucose at the lower dose. Myrtus extract did not affect the proinflammatory cytokines, expression of HSP70, HSP90, and blood physiological parameters excluding an increased basophil count. Intestinal morphology and intestinal tissue proliferation were positively affected by the *myrtus communis* supplementation. Effect of the myrtus plant on the protein and glucose metabolism should be further investigated to elucidate its potential use to improve animal health, digestion and metabolism.

## Data Availability

The original contributions presented in the study are included in the article/supplementary material, further inquiries can be directed to the corresponding author/s.
